# Cardiovascular health profiles in adolescents being born term or preterm—results from the EVA-Tyrol study

**DOI:** 10.1186/s12872-023-03360-2

**Published:** 2023-07-25

**Authors:** Christoph Hochmayr, Jean-Pierre Ndayisaba, Nina Gande, Anna Staudt, Benoit Bernar, Katharina Stock, Sophia Zollner-Kiechl, Ralf Geiger, Elke Griesmaier, Michael Knoflach, Ursula Kiechl-Kohlendorfer, Early Vascular Ageing (EVA) Study Group, Mandy Asare, Manuela Bock-Bartl, Maximilian Bohl, Christina Schreiner, Gregor Brössner, Tatjana Heisinger, Julia Klingenschmid, Martina Kothmayer, Julia Marxer, Raimund Pechlaner, Maximilian Pircher, Carmen Reiter, Stefan Kiechl, Bernhard Winder

**Affiliations:** 1 Department of Pediatrics II (Neonatology), Medical University of Innsbruck, Anichstraße 35, Innsbruck, 6020 Austria; 2 VASCage, Center on Clinical Stroke Research, Tyrol Innsbruck, Austria; 3 Department of Neurology, Medical University of Innsbruck, Anichstraße 35, Innsbruck, 6020 Austria; 4 Department of Pediatrics I, Medical University of Innsbruck, Innsbruck, Austria; 5 Department of Pediatrics III (Pediatric Cardiology, Allergology and Cystic Fibrosis), Medical University of Innsbruck, Innsbruck, Austria; 6 Department of Neurology, Hochzirl Hospital, Zirl, Austria

**Keywords:** Preterm birth, Cardiovascular health, Health metrics, Risk factors, Adolescents, Health promotion

## Abstract

**Background and aims:**

Preterm birth has been linked with an increased risk of cardiovascular (CV) disease from childhood into adolescence and early adulthood. In this study, we aimed to investigate differences in CV health profiles between former term- and preterm-born infants in a cohort of Tyrolean adolescents.

**Methods:**

The Early Vascular Aging (EVA)-Tyrol study is a population-based non-randomized controlled trial, which prospectively enrolled 14- to 19-year-old adolescents in North Tyrol, Austria and South Tyrol, Italy between 2015 and 2018. Metrics of CV health (body mass index (BMI), systolic (SBP) and diastolic blood pressure (DBP), smoking, physical activity, dietary patterns, total cholesterol and fasting blood glucose) were assessed and compared between former term- and preterm-born girls and boys.

**Results:**

In total, 1,491 study participants (59.5% female, mean age 16.5 years) were included in the present analysis. SBP and DBP were significantly higher in former preterm-born adolescents (mean gestational age 34.6 ± 2.4 weeks) compared to term-born controls (*p* < 0.01). In the multivariate regression analysis these findings remained significant after adjustment for potential confounders in all models. No differences were found in all other CV health metrics. The number of participants meeting criteria for all seven health metrics to be in an ideal range was generally very low with 1.5% in former term born vs. 0.9% in former preterm born adolescents (*p* = 0.583).

**Conclusions:**

Preterm birth is associated with elevated SBP and DBP in adolescence, which was even confirmed for former late preterm-born adolescents in our cohort. Our findings underscore the importance of promoting healthy lifestyles in former term- as well as preterm-born adolescents. In addition, we advise early screening for hypertension and long-term follow-up in the group of preterm-born individuals.

## Introduction

Preterm birth is defined as a live birth that occurs before 37 completed weeks of pregnancy [1]. About 15 million children are born prematurely worldwide every year, which corresponds to a preterm birth rate of about 11%. Rates vary by the countries´ standard of living from approximately 9% in Europe to the highest rates of over 13% in low-income countries in Asia and sub-Saharan Africa [1, 2]. According to the World Health Organization prematurity and related complications are the leading causes of mortality and morbidity in children under five years of age worldwide [3, 4]. However, due to advances in perinatal care, survival rates and survival without major morbidity have continuously improved over the past 20 years in high- and middle-income countries [5, 6]. With an increasing number of former preterm infants reaching adolescence and adulthood, new health challenges arise [7] which affect the respiratory tract, the endocrine system, and the psycho-motoric development [8–10]. In addition, prematurity has been linked to altered CV health profiles at a pre-school age [11], as well as in early adolescence and adulthood [12–14] resulting in an increased risk of CV and cardiometabolic disease [15, 16]. It has been shown that young adults born preterm are less physically active in sports in their leisure time [17, 18] or consume a more unhealthy diet than their term-born peers [19]. As these differences are modifiable by appropriate lifestyle changes, it is of great interest to identify subgroups at risk for CV and cardiometabolic disease to plan targeted intervention programs and health promotions accordingly [20]. Furthermore, there is evidence that preterm birth is associated with a higher total fat mass and more abdominal fat, which may have detrimental effects on cardiometabolic and CV health [15]. Although manifest CV and cardiometabolic disease is a rare condition in youth, its antecedents may develop early in life. There is evidence that vascular alterations like thickening of arterial vessel wall already starts in the first decades of life and is associated with classical risk factors (i.e., high BMI, elevated SBP, hypercholesterolemia or smoking) [21–23].

The aim of the present study was to investigate differences in CV health profiles between former term and preterm infants in a cohort of Tyrolean adolescents. To the best of our knowledge, we are the first to investigate the impact of preterm birth on the seven CV health metrics defined by the American Heart Association (AHA) [20] in Central Europe.

## Methods

### Study population and design

The present study was conducted as part of the EVA-Tyrol study, a population-based, non-randomized controlled trial at the Department of Pediatrics II at the Medical University of Innsbruck. EVA-Tyrol is aiming to assess the prevalence of CV health profiles and the efficacy of a health promotion intervention on CV risk factors and health behaviors in a cohort of adolescents in the whole federal province of Tyrol (Austria) with a population of about 745,000 inhabitants and in Bruneck, a city in the autonomous province of Bolzano/South Tyrol (Italy) with about 80,000 inhabitants. Local schools and big companies were contacted and pupils and apprentices with a target age between 14 and 19 years were invited to take part in this study. Whole school classes were recruited thus some older students or apprentices, who wanted to participate were also included. 2273 persons did express their interest in participation in the EVA Tyrol study, yet 171 did either not sign the informed consent or signed the informed consent but did not show up on the examination day. For the latter group no study specific measures were performed. All participants provided a written informed consent or if the participants had not attained the age of 18 years, a parent or legal guardian additionally provided written consent.

The study was designed with an intervention group, which received a baseline examination and was invited to a follow-up examination after receiving a health intervention, and a control group. For this exploratory data analysis, information from both the baseline and the control group without health intervention was used to achieve a representative cohort of the adolescent population, in the following they are referred to as the EVA-cohort. A detailed description of the study protocol has been previously published [24]. The study was performed in accordance with the Declaration of Helsinki, ethical approval was granted from the review board of the Medical University of Innsbruck, Austria (approval number AN 2015–0005 345/4.13) and all participants and legal representatives signed informed consent for participation. The study is registered at www.clinical.trials.gov(NCT number 03929692).

The present study represents a subanalysis of the EVA-Tyrol study and highlights data on the impact of preterm birth on cardiovascular health.

### Perinatal characteristics

All participating adolescents were asked bringing the so-called “mother–child booklet” with them on the day of examination. Within this official medical document health data about pregnancy, birth and regular pediatric examinations during the first 5 years of life are documented in Austria and Italy. We registered infant data on perinatal characteristics, namely gestational age (GA) and birth weight. To account for gender- and GA-specific differences, birth weight z-scores were calculated for every subject using the Fenton 2013 reference dataset [25]. Small for gestational age (SGA) was defined as birth weight < 10th percentile for GA and sex. Preterm birth was defined as being born < 37 completed weeks of gestation. Definition of early preterm was birth before 32 completed weeks of gestation, moderately preterm was defined as GA between 32–34 weeks of gestation and late preterm between 34–37 weeks of gestation.

CV health was assessed by the concept of the seven so called CV health metrics defined by the AHA including three health factors (SBP and DBP, total cholesterol and fasting blood glucose) and four health behaviors (non-smoking, BMI, regular physical activity and favorable dietary patterns) [20].

### Anthropometry

For anthropometric measurements study participants wore light indoor clothes without shoes. Weight was assessed using calibrated medical precision scales and height was determined using a Harpenden stadiometer (Holtain, Crymych, United Kingdom). BMI was calculated as body weight in kilograms divided by the square of height in meters and converted to percentiles according to reference data by Kromeyer-Hauschild et al. [26]. SBP and DBP were computed as the mean of 3 independent measurements on the left and right upper arm in a sitting position and recorded after at least 5 min at rest by an automated oscillometric device (OMRON M4-I, Omron Healthcare Co., Lake Forest, Illinois, USA). Z-scores and percentiles for SBP and DBP were calculated based on a reference data set [27].

### Assessment of lifestyle risk factors

Behavioral risk factors, such as smoking status and physical activity, were assessed in a standardized medical interview conducted by trained medical staff in attendance of a specialist in pediatrics, compiled and adapted from the questionnaires from the Atherosclerosis Risk-Factors in Male Youngsters, Atherosclerosis Risk-Factors in Female Youngsters, and Bruneck studies [21, 22, 28]. Individuals were categorized as smokers if they smoked at least one cigarette per week on a regular basis. Physical activity was recorded as participation in moderate or vigorous intensity sports (e.g., leading to an increase in heart rate and/or sweating) as average amount of minutes per day. Dietary habits were evaluated according to a score based on the Dietary Approaches to Stop Hypertension- (DASH-) diet consisting of five favorable components [29]. For meeting the criteria one point is scored for each of the following components: four to five servings of fruit and vegetable per day; at least two fish meals per week; at least three servings of whole-grain products per day, each about 30 g; less than 1.5 g of salt per day and a maximum of one liter of sugar-sweetened beverages per week, i.e., not more than 450 kcal per week, respectively.

### Blood sample collection and laboratory measurements

Blood samples were taken early in the morning after an overnight fasting of at least eight hours and were immediately stored in cooling boxes (at approximately 4 °C). After rapid transport to the ISO-certified Central Institute for Medical and Chemistry Laboratory Diagnosis at Medical University of Innsbruck serum glucose was assessed by a hexokinase method (Cobas 8000, Roche Diagnostics, Rotkreuz, Switzerland). Total cholesterol was determined by standard enzymatic colorimetric assays (Cobas 8000, Roche Diagnostics, Rotkreuz, Switzerland).

### Assessment of socio-economic status

The Family Affluence Score (FAS) was used to determine the socio-economic status. Study participants were asked about four status items (including owning a car in the family, having an own bedroom, going on vacations and number of computers in the household) and classified into the categories high, middle and low affluence [30]. Low FAS occurred in less than 1% of cases in our cohort so that it was combined with the middle affluence category for this analysis.

### Classification of education

After nine years of compulsory school attendance, the Austrian education system offers three different educational pathways. In secondary high schools adolescents receive general academic education, secondary vocational schools provide general education as well as occupation-specific knowledge or adolescents may be trained as apprentices within companies.

### Statistical analysis

Statistical analysis was performed using SPSS version 27.0 for Windows (IBM Corporation, Armonk, New York). Data of the study characteristics at baseline and follow-up are shown as mean ± SD or median (interquartile ranges) and categorical variables as count (percentages). Differences in CV health metrics were determined using t-test or Mann–Whitney-U-test (depending on data distribution) and Pearson χ^2^- test (for categorical variables). To assess the relationship between prematurity and CV health metrics in the preterm and the term group binary logistic regression with progressive adjustment was used to estimate. A stepwise approach was used with regard to covariates and potential confounders. Model A was unadjusted. Parameters included in the model were SPB or DBP, sex and age at examination in Model B, SPB or DBP, sex, age at examination, BMI, smoking status, physical activity and diet in Model C, SPB or DBP, sex, age at examination, BMI, smoking status, physical activity, diet and SGA in Model D and SPB or DBP, sex, age at examination, BMI, smoking status, physical activity, diet, SGA, fasting blood glucose and total cholesterol in Model E. *P*-values of less than 0.05 were considered statistically significant.

## Results

**Fig. 1:**
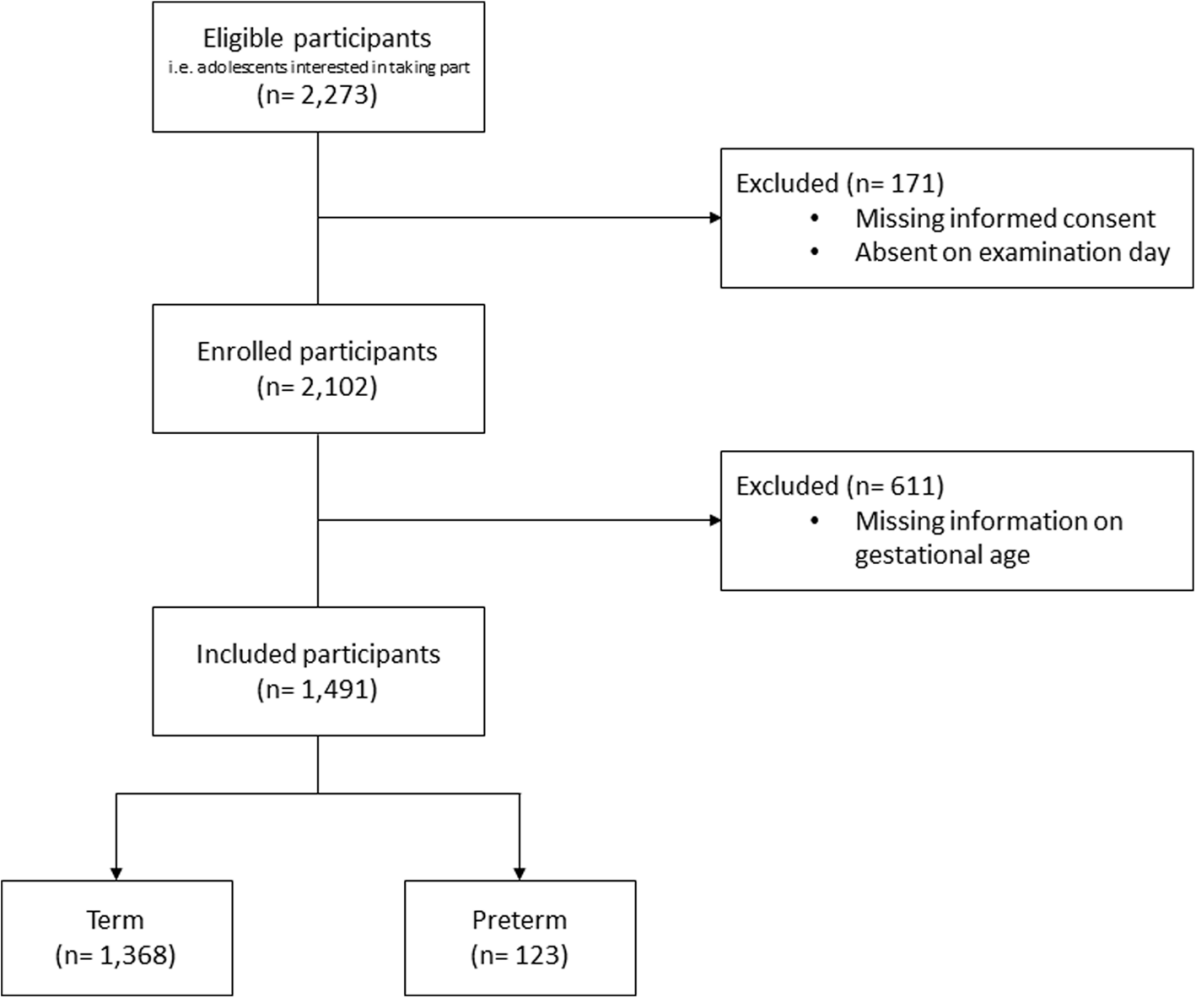
Flow chart for inclusion of study participants

A total number of 2,273 adolescents was assessed for eligibility, of which 171 were excluded due to missing informed consent or absence on examination day. Thus, 2,102 adolescents were included for the following investigation forming the EVA-cohort, with 1,573 participants in the baseline cohort and 529 adolescents who did not undergo a health intervention. Information on GA was missing in 611 cases leaving 1,491 young adults for further analysis of differences between former term- and preterm-born infants (Fig. 1).

Characteristics of the study cohort are displayed in Table 1.

**Table 1:** Characteristics of the study population

Parameter	Total population *n* = 1491	Term *n* = 1368 (91.8%)	Preterm *n* = 123 (8.2%)	*P*-value*
**Age at examination, years**	16.3 ± 1.1	16.4 ± 1.1	16.2 ± 1.1	0.177^a^
**Sex, female (%)**	887 (59.5)	819 (59.9)	68 (55.3)	0.338^b^
**FAS-Score, high (%)**	984 (66.0)	906 (66.2)	78 (63.4)	0.523^b^
**Education (%)**				
High school	507 (34.0)	468 (34.2)	39 (31.7)	0.575^b^
Vocational school	820 (55.0)	746 (54.5)	74 (60.2)	0.229^b^
Trainee	164 (11.0)	154 (11.3)	10 (8.1)	0.288^b^
**Mean gestational age, weeks**	39.4 ± 2.0	39.8 ± 1.2	34.6 ± 2.4	** < 0.01**^**c**^
**Non-smokers (%)**	1086 (72.8)	997 (72.9)	89 (72.4)	0.831^b^
**Healthy diet score, components (n)**	2.0 ± 1.1	2.0 ± 1.1	2.1 ± 1.1	0.495^a^
**Physical activity (min/day)**	53.0 (30–60)	53.1 (25–60)	52.6 (30–60)	0.362^c^
**BMI (kg/m**^**2**^**)**	21.8 ± 3.5	21.8 ± 3.5	21.5 ± 3.3	0.510^c^
**SBP (mmHg)**	122.3 11.5	122.1 ± 11.5	124.5 ± 11.7	**0.024**^**c**^
z-score Systolic BP	0.6 ± 1.1	0.6 ± 1.1	0.8 ± 1.0	**0.014**^**c**^
**DBP (mmHg)**	71.3 ± 7.6	71.1 ± 7.6	72.9 ± 7.6	**0.026**^**c**^
z-score Diastolic BP	0.2 ± 1.1	0.2 ± 1.1	0.5 ± 1.1	**0.01**^**c**^
**Total cholesterol (mg/dl)**	160.0 (138.0–177.5)	160.0 (139.0–178.0)	157.6 (137.0–175.0)	0.277^c^
**Fasting blood glucose (mg/dl)**	76.7 ± 9.4	76.7 ± 9.4	77.2 ± 9.0	0.571^a^

Values are displayed as n (%), mean ± SD, median (IQR) or %. *BMI* Body Mass Index, *DBP* diastolic blood pressure, *SBP* systolic blood pressure, *FAS* Family Affluence Score, *P values are derived from ^a^Student ´s t-test, ^b^Pearson-χ ^2^- test and ^c^Mann-Whitney-U-Test

Adolescents were categorized into the term (*n* = 1,368, 91.8%) and the preterm (*n* = 123, 8.2%) group with a mean gestational age of 39.8 (± 1.2) for the term and 34.6 (± 2.4) weeks for the preterm group, respectively. The distribution of girls and boys in the groups did not differ significantly (*p* = 0.322). No significant differences regarding socioeconomic status (*p* = 0.523) and level of education (*p* = 0.394) between the two groups of former term- and preterm-born infants were observed. In the total population, SBP (*p* = 0.024) and DBP (*p* = 0.026) were significantly higher in former preterm-born adolescents compared to term-born controls. This was also shown for SBP z-scores (*p* = 0.014) and DBP z-scores (*p* = 0.01). No differences were found in all other CV health metrics. In the multivariate binary logistic regression model higher SBP and DBP values in the preterm compared to the term population remained significant in all models that adjusted for potential confounding factors. After adjustment for sex, age at examination, BMI, smoking status, physical activity, diet, SGA, fasting blood glucose and total cholesterol a beta coefficient of -0.028 (Std. error 0.01), OR = 0.972 (95% CI: 0.953, 0.991) for SBP and a beta coefficient of -0.039 (Std. error 0.013), OR = 0.961 (95% CI: 0.937, 0.987) for DBP was observed. The results of multivariate testing are listed in Tables 2 and 3.

**Table 2:** Binary logistic regression on the effect of prematurity on systolic blood pressure

Model	Parameter	β	Std. Error	p-value	Exp (B)	95% Confidence Interval
**Model A**	**Systolic blood pressure**	**-0.018**	**0.008**	**0.028**	**0.982**	**0.967 – 0.998**
**Model B**	**Systolic blood pressure**	**-0.019**	**0.009**	**0.032**	**0.981**	**0.964 – 0.998**
	Sex	-0.002	0.209	0.991	0.998	0.663 – 1.502
	Age	0.139	0.088	0.114	1.149	0.967 – 1.365
**Model C**	**Systolic blood pressure**	**-0.025**	**0.009**	**0.009**	**0.975**	**0.957 – 0.994**
	Sex	-0.048	0.22	0.828	0.953	0.619 – 1.468
	Age	0.134	0.092	0.145	1.143	0.955 – 1.369
	BMI	0.052	0.031	0.095	1.054	0.991 – 1.121
	Smoking	-0.17	0.225	0.451	0.844	0.543 – 1.312
	Physical activity	0.002	0.003	0.42	1.002	0.997 – 1.007
	Diet	-0.71	0.09	0.427	0.931	0.781 – 1.11
**Model D**	**Systolic blood pressure**	**-0.025**	**0.009**	**0.009**	**0.976**	**0.958 – 0.994**
	Sex	-0.053	0.22	0.81	0.949	0.616 – 1.461
	Age	0.136	0.092	0.14	1.145	0.957 – 1.372
	BMI	0.054	0.032	0.089	1.055	0.992 – 1.122
	Smoking	-0.168	0.225	0.455	0.845	0.544 – 1.313
	Physical activity	0.002	0.003	0.407	1.002	0.997 – 1.007
	Diet	-0.71	0.09	0.428	0.931	0.781 – 1.11
	SGA	-0.241	0.345	0.485	0.786	0.4 – 1.545
**Model E**	**Systolic blood pressure**	**-0.028**	**0.01**	**0.004**	**0.972**	**0.953 – 0.991**
	Sex	0.026	0.248	0.918	1.026	0.631 – 1.668
	Age	0.071	0.093	0.447	1.073	0.894 – 1.289
	BMI	0.68	0.033	0.041	1.071	1.003 – 1.143
	Smoking	-0.189	0.233	0.417	0.827	0.524 – 1.308
	Physical activity	0.002	0.003	0.462	1.002	0.997 – 1.007
	Diet	-0.1	0.093	0.282	0.905	0.754 – 1.086
	SGA	-0.222	0.363	0.541	0.801	0.393 – 1.632
	Fasting blood Glucose	-0.003	0.011	0.751	0.997	0.975 – 1.018
	Total cholesterol	0.003	0.004	0.399	1.003	0.996 – 1.01

*p*-values are derived from multivariate binary logistic regression with the dependent variable being born term or preterm, β Beta Coefficient, BMI Body Mass Index, Exp (B) Odds Ratio, SGA Small for gestational age, Std. Error Standard Error

**Table 3:** Binary logistic regression on the effect of prematurity on diastolic blood pressure

Model	Parameter	β	Std. Error	*p*-value	Exp (B)	95% Confidence Interval
**Model A**	**Diastolic blood pressure**	**-0.03**	**0.012**	**0.013**	**0.97**	**0.947 – 0.994**
**Model B**	**Diastolic blood pressure**	**-0.031**	**0.012**	**0.01**	**0.969**	**0.946 – 0.993**
	Sex	-0.211	0.192	0.272	0.81	0.557 – 1.179
	Age	0.128	0.088	0.145	1.136	0.957 – 1.349
**Model C**	**Diastolic blood pressure**	**-0.034**	**0.013**	**0.007**	**0.966**	**0.943 – 0.991**
	Sex	-0.293	0.203	0.148	0.746	0.501 – 1.11
	Age	0.122	0.092	0.182	1.13	0.944 – 1.352
	BMI	0.038	0.029	0.197	1.038	0.981 – 1.1
	Smoking	-0.172	0.225	0.446	0.842	0.542 – 1.309
	Physical activity	0.001	0.003	0.648	1.001	0.996 – 1.006
	Diet	-0.08	0.09	0.372	0.923	0.774 – 1.1
**Model D**	**Diastolic blood pressure**	**-0.034**	**0.013**	**0.007**	**0.966**	**0.943 – 0.991**
	Sex	-0.298	0.203	0.141	0.742	0.499 – 1.104
	Age	0.125	0.092	0.174	1.133	0.946 – 1.356
	BMI	0.04	0.029	0.178	1.04	0.982 – 1.102
	Smoking	-0.17	0.225	0.449	0.843	0.543 – 1.311
	Physical activity	0.001	0.003	0.631	1.001	0.996 – 1.006
	Diet	-0.08	0.09	0.374	0.923	0.775 – 1.101
	SGA	-0.264	0.346	0.444	0.768	0.39 – 1.511
**Model E**	**Diastolic blood pressure**	**-0.039**	**0.013**	**0.003**	**0.961**	**0.937 – 0.987**
	Sex	-0.252	0.229	0.272	0.777	0.496 – 1.218
	Age	0.057	0.093	0.538	1.059	0.883 – 1.271
	BMI	0.051	0.031	0.101	1.052	0.99 – 1.118
	Smoking	-0.191	0.234	0.413	0.826	0.523 – 1.306
	Physical activity	0.001	0.003	0.717	1.001	0.996 – 1.006
	Diet	-0.107	0.093	0.248	0.898	0.748 – 1.078
	SGA	-0.243	0.364	0.505	0.784	0.384 – 1.602
	Fasting blood Glucose	-0.002	0.011	0.864	0.998	0.977 – 1.02
	Total cholesterol	0.004	0.004	0.295	1.004	0.997 – 1.011

*p*-values are derived from multivariate binary logistic regression with the dependent variable being born term or preterm, β Regression Coefficient B, BMI Body Mass Index, Exp (B) Odds Ratio, SGA Small for gestational age, Std. Error Standard Error

**Fig. 2:**
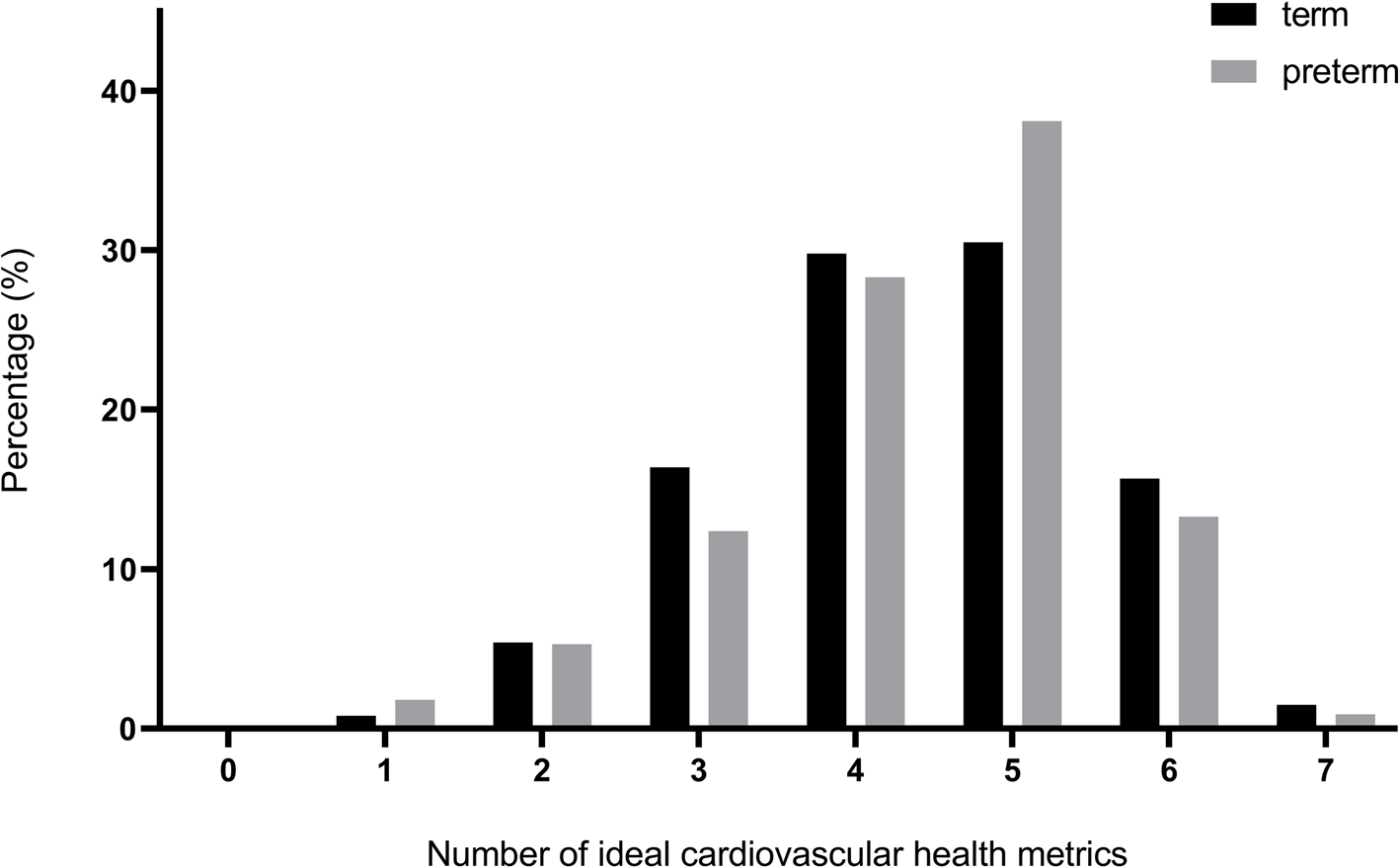
Prevalence of ideal cardiovascular health determinants in former term- and preterm-born adolescents. Displays the percentages of adolescents by number of ideal cardiovascular health metrics according to being born term or preterm

Figure 2 shows the number of health metrics in which participants were able to achieve the ideal range. Only in 1.5% of former term-born adolescents and 0.9% and former preterm-born adolescents all seven CVH determinants were in an ideal range. There was no significant difference in the distribution of health metrics classified as ideal between former term- and preterm-born infants (*p* = 0.583).

## Discussion

Although preterm birth rates in developed high-income countries have stagnated or declined over the past 1.5 decades [31], prematurity remains a substantial health determining factor in the pediatric population. Medical advances have led to an increasing number of prematurely born children reaching adolescence and adulthood without major damage [5, 6] and a growing body of literature now focusses on long-term implications of preterm birth. Morbidity and mortality in preterm-born adolescents are increased and the associated costs are a socioeconomic issue [32]. In this regard, a key aspect is the evidence of an increased risk of CV disease for former preterm-born individuals [11–14, 16]. To achieve the AHA´s goals of reducing CV disease, myocardial infarction, and stroke, it is necessary to identify markers of increased CV risk in order to organize effective targeted preventive measures [20]. Therefore, the aim of the present study was to assess CV health metrics (SBP and DBP, BMI, smoking, physical activity, dietary patterns, total cholesterol and fasting blood glucose) and to determine differences between former term- and preterm-born adolescents from North- and South Tyrol, a region in the high-income countries Austria and Italy. Results of our study confirm the findings of previous observations but further underscore the impact of prematurity on CV risk.

Regarding blood pressure, SBP as well as DBP were significantly higher in former preterm-born adolescents compared to term-born controls after adjustment for sex, age at examination, BMI, smoking status, physical activity, diet, being SGA, fasting blood glucose and total cholesterol in the multivariate logistic regression model. This finding is corroborated by several meta-analyses, which have also demonstrated an increased risk for elevated blood pressure in the preterm population [13, 33, 34] and has also been described for former very preterm-born children (mean gestational age 29.3 weeks) at preschool age and for late preterms (mean gestational age 34.8 weeks) in adolescence by our study group previously [11, 35].

Although these differences are small and within normal ranges for this age group, there is evidence that elevated blood pressure levels associated with preterm birth worsen with increasing age [36] and may be responsible for an increased risk of CV diseases in later life [13, 16].

In addition to prenatal factors, such as preeclampsia, gestational diabetes, obesity, intrauterine growth retardation, which lead to adverse fetal programming, there is evidence that a number of other causes play a role in the development of elevated blood pressure in former preterm infants during the postnatal course [13, 33, 37], for instance impaired nephrogenesis, extra uterine growth restriction or forced weight gain caused by high calorie nutrition [13].

Regarding all other health metrics, we could not identify any impact of preterm birth in our cohort. Altered lipid profiles [38] as well as pathologic glucose metabolism [39] have been identified as potential cardiometabolic risk factors in former preterm-born adolescents. However, findings are inconsistent and a relation to the extent of prematurity with the highest risk in the extremely preterm-born ones has been found [40]. Several studies have reported an impact of prematurity on physical activity in later life with extremely preterm ones being less active than their term-born peers [17, 18]. A mean gestational age of 34 weeks in our cohort may explain why no significant difference between former preterm-born and term-born adolescents regarding lipid and glucose metabolism and physical activity was found. In addition, sports are very much promoted in the Tyrol region, which means that also at-risk groups such as former premature individuals can be motivated by the number of activities offered. In addition, the impact of preterm birth was also shown for dietary habits with less healthy dietary quality and behaviors in young adults born prematurely [19, 41]. We could also not show any difference regarding dietary habits according to the DASH-score in the current study, but it has to be added that the rate of healthy diet in our cohort is generally very low with more than 30% categorized as poor in both the preterm as well as in the term group. In general, our results show that there is potential for improvement in the prevalence of ideal CV health in both groups in our cohort, because only 0.9% of former preterm-born and 1.5% of term-born teenagers met ideal criteria for all seven health metrics defined by the AHA. For example, the proportion of smokers in both groups is to be regarded as relatively high at about 30%. This highlights the need of specialized health interventional programs in adolescence in order to sustain improvements in CV health behavior that persist into adulthood.

### Strengths and limitations

One of the main strengths of our study is the large and homogenous study cohort, which includes adolescents from all types of schools and apprentices of the same age from the entire study region. Furthermore, we investigated a broad spectrum of CV health parameters and data was collected prospectively by a small and stable study team within approximately three years.

The small number of former preterm-born adolescents compared with term-born individuals is a limitation, which must be mentioned. The rate of former preterm born adolescents in our cohort, however, corresponds to the rate of preterm birth in Austria [42]. Unfortunately, 611 individuals had to be excluded from analysis because of missing information on GA. The main reasons were either that the mother–child booklet was no longer available or lack of consent of the mother. Due to the small number of preterm infants and consequently the low number of former early preterm (< 32 weeks of gestation, *n* = 12) and moderate preterm (32–34 weeks of gestation, *n* = 21) adolescents further sub-group analyses could not be performed. According to recent literature hereditary factors may play an important role in developing hypertension [43]. Unfortunately, no data were available on the blood pressure levels of the participants´ parents in our study. Furthermore, measurement of body impedance was not part of the study protocol thus we cannot draw any conclusions about the differences in body fat composition between former preterm- and term-born adolescents.

### Implications and conclusion

The results of our study underscore the impact of prematurity on CV risk. In our cohort, SBP and DBP were significantly higher in former preterm-born than in term-born adolescents. It should be emphasized that elevated blood pressure levels were even found in adolescents born late preterm. Therefore, early screening for hypertension and long-term follow-up in individuals with a history of preterm birth is warranted. The low number of adolescents meeting the criteria for ideal CV health metrics highlights the need for promotion of a healthy lifestyle in both former preterm- and term-born individuals.

## Data Availability

The data that support the findings of this study are available from the corresponding author upon reasonable request.

## Abbreviations

AHA: American Heart Association
BMI: Body Mass Index
CV: Cardiovascular
DASH: Dietary Approaches to Stop Hypertension
DBP: Diastolic Blood Pressure
EVA: Early Vascular Ageing
FAS: Family Affluence Score
GA: Gestational Age
SBP: Systolic Blood Pressure
SGA: Small for Gestational Age
